# Novel Biomarkers for SARS-CoV-2 Infection: A Systematic Review and Meta-Analysis

**DOI:** 10.3390/jpm15060225

**Published:** 2025-06-01

**Authors:** Sara Weronika Snopkowska Lesniak, Diego Maschio, Fernando Neria, Beatriz Rey-Delgado, Victor Moreno Cuerda, Cesar Henriquez-Camacho

**Affiliations:** 1 Servicio de Medicina Interna, Hospital Universitario de Mostoles, 28935 Madrid, Spain; saraweronika.snopkowska@salud.madrid.org (S.W.S.L.); diego.maschio@salud.madrid.org (D.M.); vmcuerda@salud.madrid.org (V.M.C.); 2 Facultad de Medicina, Universidad Francisco de Vitoria, 28223 Madrid, Spain; fernando.neria@ufv.es (F.N.); b.rey@ufv.es (B.R.-D.); 3 Facultad de Ciencias de la Salud, Universidad Rey Juan Carlos, 28922 Madrid, Spain

**Keywords:** biomarkers, SARS-CoV-2, COVID-19, MR-proADM, KL-6

## Abstract

**Background:** COVID-19, caused by SARS-CoV-2, has posed significant challenge to global healthcare systems, necessitating reliable biomarkers to predict disease severity and mortality. This systematic review and meta-analysis evaluated the prognostic value of novel biomarkers in COVID-19 patients. The aim of this study was to identify and prioritize the most prognostically relevant novel biomarkers associated with COVID-19 outcomes. **Methods:** We conducted a systematic review and meta-analysis of the available evidence. A systematic search of PubMed and Web of Science was performed to identify studies on the COVID-19 biomarkers. Observational studies that compared poor (severe disease/mortality) and good outcomes were included. For continuous measures, standard mean differences (SMDs) with 95% confidence intervals (CIs) were calculated. Pooled sensitivity, specificity, diagnostic odds ratio (DOR), and summary receiver operating characteristic (SROC) curve analyses for the biomarkers were used. The risk of bias was assessed using the Newcastle–Ottawa scale. **Results:** Of the 2907 screened studies, 38 were included (21 in the meta-analysis). MR-proADM showed higher levels of prediction for poor outcomes (SMD = 1.40, 95% CI: 1.11–1.69; AUC 0.74–0.96; sensitivity, 85%; specificity, 71%). The neutrophil-to-lymphocyte ratio (NLR) showed a high correlation with disease severity (SMD = 1.07, 95% CI: 0.79–1.35; AUC 0.73–0.98; sensitivity, 86%; specificity, 78%). Increased KL-6 levels were associated with lung injury (SMD = 1.22, 95% CI: 0.24–2.19; AUC 0.85–0.95). Other biomarkers (suPAR, miR-155, Galectin-3) showed promise but lacked sufficient data for pooled analysis. Heterogeneity was observed among the included studies in terms of diagnostic accuracy. These findings indicate that elevated levels of MR-proADM, NLR, and KL-6 are significantly associated with COVID-19 prognostic accuracy to guide patient management. **Conclusions:** MR-proADM, NLR, and KL-6 levels demonstrated strong prognostic value for COVID-19 severity and mortality. These biomarkers can enhance clinical decision-making.

## 1. Introduction

In 2019, SARS-CoV-2 emerged in Wuhan, China, and spread worldwide, causing a pandemic responsible for more than 768 million cases and almost 7 million deaths globally [1]. Severe acute respiratory syndrome coronavirus 2 (SARS-CoV-2) is the etiologic agent of the current global coronavirus disease 2019 (COVID-19) pandemic [2]. COVID-19 presents as an asymptomatic or mild-to-severe-to-lethal form. Mild COVID-19 cases are characterized by flu-like symptoms, such as fever, monoarthralgia, cough, and nasal congestion [3]. Severe COVID-19 cases are characterized by acute respiratory distress syndrome (ARDS) triggered by a hyper-inflammatory response known as a cytokine storm, with respiratory failure, multiorgan disease, and death [4]. Patients with ARDS have a persistent elevation of inflammatory cytokines (IL-6, IL-8, and TNF-α) and are probably associated with disease severity and prognosis [5,6].

In this setting, clinical findings and analytical parameters are crucial for identifying high-risk patients, providing better treatment, and improving clinical outcomes [7,8]. During the COVID-19 pandemic, extensive evidence has been produced, and new biomarkers have been described, linking the roles of cytokines, traps, and nucleosomes [9]. Despite progress in treatment and prevention, immune mechanisms and host responses remain unclear [10]. Several biomarkers have been associated with the severity of COVID-19, including D-dimer, C-reactive protein, ferritin, and lactate dehydrogenase. However, their use has not yet been tested in a clinical setting [11].

New biomarkers such as Mid-Regional pro-Adrenomedullin (MR-proADM), neutrophil-to-lymphocyte ratio (NLR), and Krebs von den Lungen (KL-6) have been tested in different settings with better specificity than conventional markers [12]. MR-proADM is a vasoactive peptide linked to endothelial dysfunction and organ failure. Elevated levels are correlated with severe COVID-19, ARDS, and mortality [13]. The NLR is a simple and cost-effective marker of systemic inflammation. This reflects immune dysregulation, where high neutrophils and low lymphocytes predict worse outcomes [14]. KL-6 is a glycoprotein produced by damaged lung epithelial cells and is strongly associated with pulmonary fibrosis and severe respiratory failure in COVID-19 [15]. Other emerging biomarkers, such as the soluble urokinase Plasminogen Activator Receptor (suPAR), MicroRNA-155 (miR-155), and Galectin-3, indicate immune activation and hyperinflammation. miR-155 is a regulator of inflammatory signaling pathways that are potentially linked to cytokine storms [16]. Galectin-3 is involved in fibrosis and chronic inflammation, which may predict long-term lung damage [17].

Given the numerous studies published on novel biomarkers for COVID-19, there is a critical need to synthesize and evaluate the clinical relevance of these findings. Although these biomarkers show promise, their clinical utility remains understudied in large-scale meta-analyses. This systematic review and meta-analysis aimed to identify and prioritize the most prognostically relevant novel biomarkers associated with COVID-19 outcomes, quantify their strength of correlation with clinical endpoints, and assess their potential utility in guiding risk stratification and patient management strategies.

## 2. Material and Methods

This study was conducted in accordance with the Preferred Reporting Items for Systematic Review and Meta-Analysis (PRISMA) statement [18] and was registered in the PROSPERO International Prospective Register of Systematic Reviews (No. CRD42024594709).

### 2.1. Inclusion and Exclusion Criteria

We chose the following studies for this review: observational studies regarding biomarkers in COVID-19 adult patients, comparing poor and good outcomes. There were no restrictions on the type of study (RCT, quasi-randomized trials, or case reports). Reviews, case reports, guidelines, systematic reviews, meta-analyses, and animal studies were also excluded.

### 2.2. Search Strategy

We searched two electronic databases, PubMed and Web of Science, through July 2024, using relevant keywords. WOS: (TS = (biomarkers) AND ((TS = (“COVID-19” OR “SARS-CoV-2”)) AND TS = (prognosis OR diagnosis))) AND DT = (Article). PubMed: (((biomarkers [MeSH Terms]) OR (biomarkers [Title/Abstract])) AND (“COVID-19” [Title/Abstract] OR “SARS-CoV-2” [Title/Abstract])) AND ((prognosis OR diagnosis [MeSH Terms]) OR (prognosis [Title/Abstract] OR diagnosis [Title/Abstract])).

### 2.3. Selection of Studies

Eligibility screening was conducted based on our inclusion criteria in two steps. First, we screened titles and abstracts for eligibility and then retrieved and screened the full-text articles of the selected abstracts for inclusion in this review. Two reviewers (F.N. and B.R.) independently conducted the literature search, screening, and retrieved trials. They screened the titles and abstracts. Following this preliminary screening, S.S. and D.M. retrieved full reports of potentially relevant trials and applied the inclusion criteria to the full reports using an eligibility form. If the eligibility was not clear, the trial authors were contacted for clarification. We scrutinized eligible trials to ensure that each trial was included only once. We listed the trials that were not eligible for inclusion and explained the reasons for their exclusion.

### 2.4. Data Extraction and Quality Assessment

We collected data from the included studies using a standard data extraction template by a pair of distinct reviewers (S.S. and D.M.), and the CHC arbitrated any conflicts. The domains of the extracted data included the number of patients, year of publication, country of study, biomarker level, and outcomes. Two reviewers (S.S. and D.M.) independently assessed the risk of bias for each study. The quality of each study was categorized using the Newcastle–Ottawa scale for cohort and case-control studies by group selection (0–4 points), comparability (0–2 points), and exposure/outcome reliability (0–3 points) [19]. Studies were categorized as good quality if they scored ≥7 points, fair quality if they scored 5–6 points, and poor quality if they scored <5 points.

### 2.5. Statistical Analysis

Statistical analysis was performed using the RevMan 5.4 software (Nordic Cochrane Center, Cochrane Collaboration, Copenhagen, Sweden) and STATA 12. For continuous measures, standard mean differences (SMDs) with 95% confidence intervals (CIs) were calculated. When the continuous outcome was reported in a study as median, range, and interquartile range, the means and variances were calculated using the formula described by Wan et al. [20]. We used the χ^2^ test to assess the heterogeneity between studies. Statistical significance was set at *p* < 0.05, and a random-effects model was used. I^2^ tests were used to assess the degree of heterogeneity and were interpreted as follows: 0–25% might be important, 26–50% indicates moderate heterogeneity, 51–75% suggests substantial heterogeneity, and 76–100% reflects considerable heterogeneity [21]. Otherwise, a fixed-effects model was used to pool outcomes. Outcomes reported in these studies were included in the meta-analysis. Sensitivity analysis, excluding one study at a time, was used to explore the source of heterogeneity in the analyses. Publication bias among the studies was investigated by visual inspection of the funnel plot and quantitative analysis using the Egger’s test. A random effects model was used to calculate the pooled sensitivity, specificity, and diagnostic odds ratio (DOR) as a biomarker to predict poor clinical outcome in COVID-19 patients. We constructed a summary receiver operating characteristic (SROC) curve analysis by plotting the summary points of sensitivity and specificity.

We summarized the values of the area under the receiver operating characteristic curve as well as the sensitivity and specificity of the included studies.

## 3. Results

**Figure 1:**
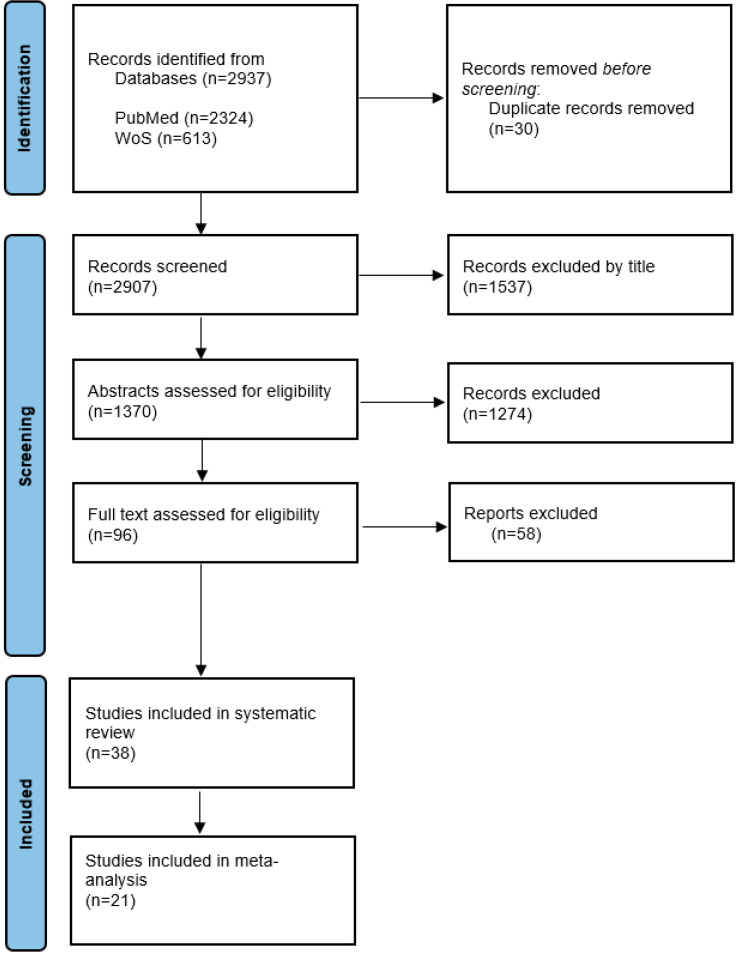
Systematic reviews and meta-analysis flow diagram.

Of the 2907 papers initially retrieved for screening, 96 were eligible for full-text screening, and 58 were excluded. Thus, 38 studies were included (Figure 1).

### 3.1. Characteristics of Included Studies

A total of 2937 studies were retrieved through the initial search (Figure 1), of which 30 duplicates were removed. After screening the titles of the remaining articles, 1537 articles were excluded. The abstracts of the remaining 1370 studies were analyzed based on the inclusion and exclusion criteria, and after excluding 1274 studies, the full texts of 96 studies were assessed. Finally, 38 studies were selected for systematic review, and 21 studies were included in the meta-analysis. Seventeen studies were excluded from the meta-analysis because of a lack of available data.

The baseline characteristics of the included studies are summarized in Table 1.

### 3.2. Biomarkers

#### 3.2.1. MR-proADM

**Figure 2:**
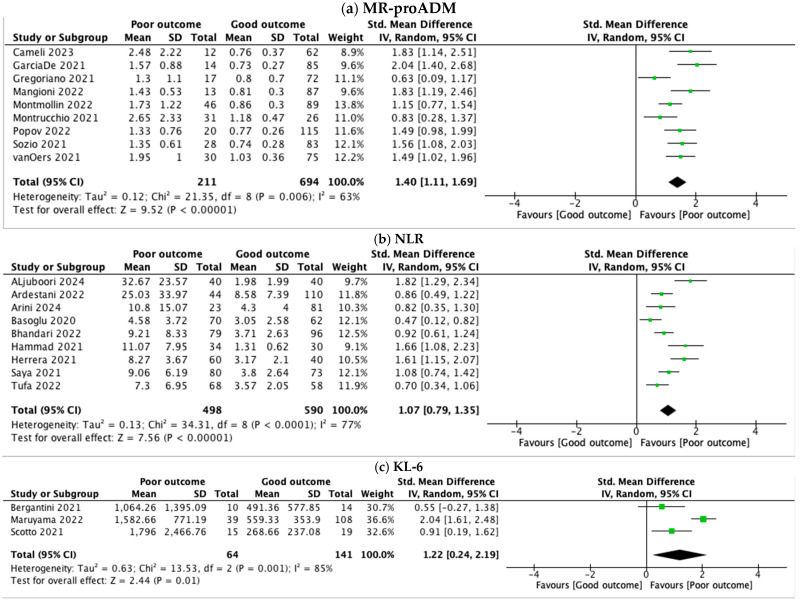
Forest plots of the meta-analysis of MR-proADM, NLR, and KL-6, pooled by standard mean difference (SMD) and confidence interval (CI). (**a**) MR-proADM levels were 1.4 SMD higher in severe COVID-19 (95% CI: 1.11–1.69), with substantial heterogeneity (I^2^ = 63%). (**b**) NLR was 1.07 SMD higher in patients with severe COVID-19 (95% CI: 0.79–1.35), with considerable heterogeneity (I^2^ = 77%). (**c**) KL-6 levels were 1.22 SMD higher in severe COVID-19 (95% CI: 0.24–2.19), with considerable heterogeneity (I^2^ = 85%).

Nine studies [38,39,40,41,42,43,44,45,46] involving 905 patients (211 with poor outcomes vs. 694 with good outcomes) were investigated. One study was excluded because of a lack of statistical data [37]. The pooled standard mean difference between the two groups was 1.40 (95% CI 1.11, 1.69), showing a strong association between higher levels of MR-proADM and poor outcome in COVID-19 patients (Figure 2a). The AUC was 0.74–0.96, showing a high diagnostic accuracy. The heterogeneity was substantial among the studies (I^2^ = 63%).

#### 3.2.2. NLR

Regarding NLR, we reviewed nine studies [22,23,24,25,27,28,29,30,31] with a total of 1088 patients (498 with poor outcome vs. 590 with good outcome). One study was excluded because of a lack of statistical data [26]. NLR was higher in patients with poor outcomes (SMD 1.07, 95% CI 0.79, 1.35) (Figure 2b), showing a significant correlation with severity. The AUC was 0.73–0.98, which shows a robust predictive performance. The heterogeneity between studies was substantial (I^2^ = 77%).

#### 3.2.3. KL-6

We analyzed KL-6 levels in 205 patients (64 with poor outcomes vs. 141 with good outcomes) from three studies [34,35,36]. Two studies lacked statistical data [32,33]. As shown in Figure 2c, the pooled standard mean difference between the two groups indicated a significant increase in KL-6 levels among patients with poor outcome (SMD 1.22, 95% CI 0.24, 2.19). As for the other biomarkers evaluated, the heterogeneity was substantial (I^2^ = 85%).

#### 3.2.4. Other Biomarkers: Amyloid A, miR-155, Galectin-3, and SuPAR

We were unable to assess the standard mean difference for the other four biomarkers evaluated due to a lack of data: Amyloid A [47,48,49], miR-155 [50,51,52], Galectin-3 [53,54], and suPAR [55,56,57,58,59].

#### 3.2.5. Receiver Operating Curve Analysis for New Biomarkers as Predictors of Poor Outcome in COVID-19 Patients

**Figure 3:**
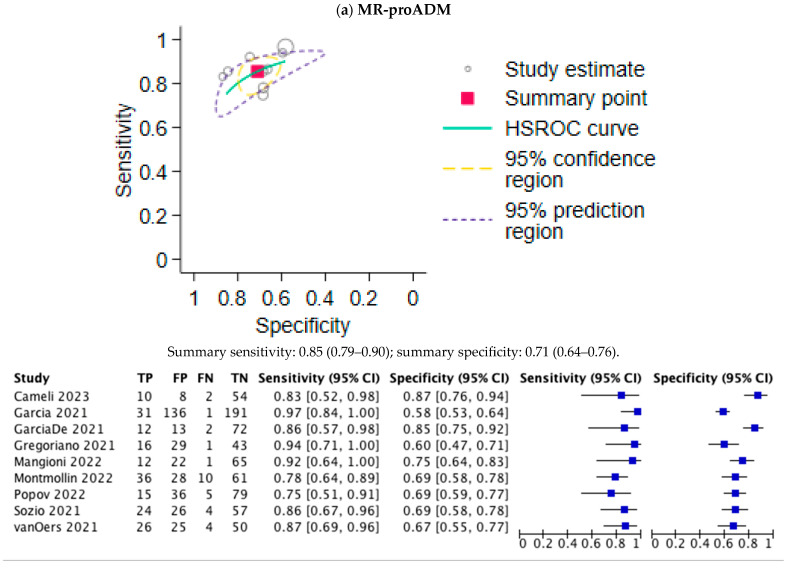

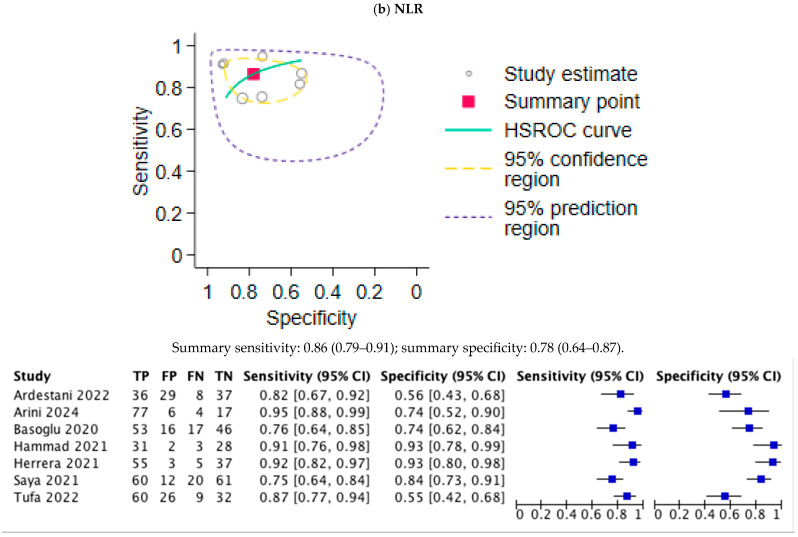
Summary receiver operating characteristic (SROC) curves of the meta-analysis of MR-proADM and NLR. TP, true positive; FP, false positive; FN, false negative; TN, true negative. (**a**) Summary AUC for MR-proADM was 0.85 (95%CI: 0.74–0.96) with optimal cut-off at 1.01 nmol/L (sensitivity 85%, specificity 71%). (**b**) Summary AUC for NLR was 0.86 (95%CI: 0.79–0.91) with optimal cut-off at 6.4 (sensitivity 86%, specificity 78%).

The values of the area under the ROC curve, sensitivity, and specificity are summarized in Table 2. The ROC curves for the biomarkers MR-proADM and NLR are shown in Figure 3a and Figure 3b, respectively. The pooled sensitivity and specificity of MR-proADM were 0.85 (95% CI, 0.79–0.90) and 0.71 (95%CI, 0.64–0.76), respectively, showing they are effective for risk stratification. The DOR was 22.71 (95%CI, 8.97–57.48). The pooled sensitivity and specificity of NLR were 0.86 (0.79–0.91) and 0.78 (0.64–0.87), respectively, showing that they are useful for the early detection of high-risk patients.

### 3.3. Quality Assessment

The included studies were evaluated using the Newcastle–Ottawa scale. Most studies (26 of 38, 68.4%) scored >7, indicating good quality and supporting reliability. Seven studies (18.4%) scored 5–6 (fair quality), often because of inadequate control for confounders, and five studies (13.2%) scored <5 (poor quality), primarily because of unclear outcome ascertainment or small sample sizes. A low risk of bias (>7 points) dominated the pooled analysis (MR-proADM and NLR). Heterogeneity was noted (I^2^ = 63–85%), but sensitivity analyses confirmed robustness.

## 4. Discussion

We investigated the diagnostic accuracy of new biomarkers for predicting poor outcomes in patients with severe COVID-19. This systematic review and meta-analysis identified MR-proADM, NLR, and KL-6 as robust predictors of severe COVID-19 outcomes, with AUC values exceeding 0.80 for mortality and ICU admission. Our findings extend beyond conventional biomarkers (e.g., CRP, D-dimer, and ferritin) by highlighting the pathophysiology-specific markers of endothelial dysfunction (MR-proADM), immune dysregulation (NLR), and lung injury (KL-6). One of the key aspects of COVID-19 is the body’s inflammatory response, which plays a crucial role in determining the severity of the illness. In this setting, identifying inflammatory biomarkers is fundamental for predicting the disease severity and patient prognosis.

Many studies have demonstrated the association between disease severity and the most common inflammation biomarkers, such as C-reactive protein (CRP), ferritin, interleukin-6 (IL6), and D-dimer. However, several novel biomarkers have been proposed and analyzed during the pandemic. In this article, we review the literature on seven new biomarkers studied in COVID-19 and their association with patient outcomes.

MR-pro-Adrenomedullin (MR-proADM) is a biomarker that has emerged as a potential indicator of organ failure and poor prognosis in COVID-19 patients. It is produced by endothelial cells and reflects overall vascular health. Elevated levels of MR-proADM have been associated with severe disease outcomes, making it a valuable tool for risk stratification in hospitalized COVID-19 patients. Rapid assessment can aid clinicians in identifying individuals at a higher risk of complications, thereby optimizing patient management. Cameli et al. [39] identified MR-proADM as a significant prognostic biomarker for COVID-19 patients. Their study found that patients with MR-proADM levels ≥1.02 nmol/L at admission had a markedly higher mortality rate (39.1%) than those with lower levels of proADM (2.2%). This demonstrates the effectiveness of this biomarker in predicting in-hospital mortality and the need for respiratory support, making it a crucial tool for clinical decision-making in COVID-19 management. García et al. [40] conducted a study in Spain, where they highlighted the prognostic value of MR-proADM in COVID-19 patients. Their findings showed that MR-proADM levels above 1.01 nmol/L had the highest performance in predicting 28-day mortality, with an area under the curve (AUC) of 0.905. Additionally, MR-proADM was independently associated with a 10.47 times higher risk of mortality (hazard ratio [HR]: 10.470) and 6.803 times higher risk of progression to severe disease (HR: 6.803). These results underscore the role of biomarkers in early prognostic assessment and clinical decision-making.

In accordance with the previously mentioned studies, our meta-analysis showed a significant association between higher levels of MR-proADM and poor outcome in COVID-19 patients. The biomarker has good sensitivity (85%) and moderate specificity (71%) for predicting poor outcomes, with an AUC of 0.74–0.96 across studies. Our meta-analysis (SMD = 1.40, AUC 0.74–0.96) confirmed its role in endothelial injury and multiorgan failure. Unlike CRP, MR-proADM reflects microvascular leakage, which is a hallmark of severe COVID-19. Thus, MR-proADM may be useful for risk stratification in hospital settings owing to its strong correlation with disease severity and mortality.

The neutrophil-to-lymphocyte ratio (NLR) has been identified as an indicator of inflammation and disease severity in COVID-19 patients. An increased NLR reflects an exaggerated immune response, characterized by an increase in neutrophils (inflammatory markers) and decrease in lymphocytes (key for adaptive immunity). This biomarker has proven useful in distinguishing between mild and severe cases, suggesting that elevated levels may predict a poor disease prognosis. Saya et al. [30] identified NLR to be able to predict mortality with high accuracy, with a 7.4 cut-off value, while Tufa et al. [31] stated that NLR is a biomarker with only modest accuracy for predicting disease severity and mortality. Our data are in line with those of previous studies, highlighting a positive correlation between NLR and poor outcomes. This method showed high sensitivity (86%) and specificity (78%), with AUC values ranging from 0.73 to 0.98. A cut-off value >6.4 supports its use even in resource-limited settings. Its rise precedes clinical deterioration by 48–72 h, offering a time window for intervention. The clinical utility of this biomarker is easy, and cost-effective, and can aid in the early identification of high-risk patients.

KL-6 (Krebs von den Lungen-6) is a glycoprotein biomarker associated with lung epithelial damage. In COVID-19 patients, elevated KL-6 levels indicate severe lung involvement, particularly in those requiring mechanical ventilation. This biomarker helps predict disease severity and potential development of pulmonary fibrosis, making it useful for the early identification of high-risk patients and monitoring lung recovery over time. Alessandro et al. conducted two studies, showing that increased KL-6 serum concentrations are associated with disease severity [33] and with fibrotic lung alterations [32]. Similarly, Maruyama et al. [35] found that the peak KL-6 value had precise accuracy in the discrimination of patients with poor prognosis when using a cut-off of 966 U/mL cut-off. According to our meta-analysis, higher KL-6 levels were a predictive factor of poor outcomes in hospitalized COVID-19 patients, with AUC values ranging from 0.85 to 0.95. This biomarker may be particularly valuable for assessing lung injury and progression to ARDS in COVID-19 patients.

Limited data precluded meta-analysis for other biomarkers (Amyloid A, miR-155, Galectin-3, and suPAR), but individual studies suggested diagnostic potential, particularly for suPAR (AUC: 0.71–0.81) and miR-155 (AUC: 0.91–0.93).

There are some limitations of our systematic review: There were differences in the type of studies (prospective or retrospective), inclusion and exclusion criteria, patients used, and cut-off values of included studies; also, pooled data were only used for analysis. In addition, there was significant heterogeneity (I^2^: 63–85%) between the included studies for diagnostic accuracy, which might be caused by the characteristics of the included patients, study designs, different control groups, and outcome definitions.

## 5. Conclusions

Inflammation is a hallmark of COVID-19 and contributes to disease severity and poor outcomes. Along with classical biomarkers, many novel inflammatory biomarkers have been investigated during the pandemic. Higher MR-proADM, NLR, and KL-6 levels were associated with an increased risk of ICU admission and death. These are validated biomarkers for predicting COVID-19 severity and mortality. These biomarkers may complement traditional markers (CRP, D-dimer, and ferritin), offering superior prognostic precision by targeting distinct disease mechanisms and improving clinical decision making. This study provides novel evidence for new biomarkers with a strong prognostic value for COVID-19. Their integration into clinical practice could improve risk stratification, therapeutic decisions, and patient outcomes by enabling early intervention and personalized care. These findings highlight the importance and need for large-scale multicenter studies to validate novel biomarker performance and cut-off values, which can help better diagnose and assess standardized protocols and prognosis in inflammatory diseases.

## Figures and Tables

**Table 1:** Characteristics of included studies.

Study ID	Country	Year	Sample	Severity Definition	Design	Poor Outcome			Good Outcome			Biomarker	NOS Scale
						No	Age	Female%	No	Age	Female%		
AlJuboori 2024 [22]	Iraq	2021	160		Case-control observational study	40	49	-	40	49	-	NLR	7
Ardestani 2022 [23]	Iran	2020	209	In-hospital mortality	Retrospective observational	44	-	-	110	-	-	NLR	7
Arini 2024 [24]	Indonesia	2022	-	Disease severity	Cross-sectional observational study	23	-	74%	81	-	69%	NLR	6
Bhandari 2022 [25]	India	2020	175	Disease severity	Retrospective observational study	79	48	20%	96	38	25%	NLR	6
Bohra 2022 [26]	-	-	-	Disease severity	-	-	-	-	-	-	-	NLR	-
Hammad 2021 [27]	Egypt	-	64	Disease severity	Prospective observational study	34	-	-	30	-	-	NLR	-
Basoglu 2020 [28]	Turkey	2020	132	Disease severity	Single-center observational study	70	64	54%	62	51	42%	NLR	8
Herrera 2021 [29]	Tukey	-	100	Disease severity	Prospective case-control study	60	66	63%	40	64	55%	NLR	7
Saya 2021 [30]	Algeria	2020	153	Disease severity	Prospective observational study	80	65	29%	73	57	37%	NLR	8
Tufa 2022 [31]	Ethiopia	2020	126	In-hospital mortality	Prospective cohort study	68	60	43%	58	32	43%	NLR	8
Alessandro 2021 [32]	Italy	2020	60	Disease severity	Prospective observational study	14	62	14%	12	64	42%	KL-6	7
Alessandro 2020 [33]	Italy	2020	22	Disease severity	Prospective observational study	12	62	25%	10	64	40%	KL-6	7
Bergantini 2021 [34]	Italy	2020	41	Disease severity	Single-center observational study	10	65	20%	14	62	21%	KL-6	8
Maruyama 2022 [35]	Spain	2021	147	Disease severity	Retrospective observational study	39	79	-	108	69	-	KL-6	8
Scotto 2021 [36]	Italy	2021	34	In-hospital mortality	Prospective observational study	15	-	-	19	-	-	KL-6	8
Garcia 2021 [37]	Spain	2020	359	In-hospital mortality	Prospective observational study	32	76	34%	327	57	36%	MR-proADM	8
Gregoriano 2021 [38]	Switzerland	2020	89	In-hospital mortality	Prospective observational study	17	-	-	72	67	35%	MR-proADM	8
Cameli 2023 [39]	Italy	2020	74	In-hospital mortality	Prospective observational study	12	86	-	62	65	-	MR-proADM	7
GarciaDe 2021 [40]	Spain	2020	99	In-hospital mortality	Prospective observational study	14	76	29%	85	64	40%	MR-proADM	8
Montmollin 2022 [41]	France	2021	135	In-hospital mortality	Prospective observational study	46	71	28%	89	58	-	MR-proADM	8
Mangioni 2022 [42]	Italy	2021	100	In-hospital mortality	Prospective observational study	13	77	39%	87	63	36%	MR-proADM	8
Montrucchio 2021 [43]	Italy	2020	57	In-hospital mortality	Prospective observational study	31	67	10%	26	59	15%	MR-proADM	8
Popov 2022 [44]	Russia	2020	135	In-hospital mortality	Prospective observational study	20	73	-	115	62	-	MR-proADM	8
Sozio 2021 [45]	Italy	2020	111	In-hospital mortality	Retrospective observational study	28	-	-	83	-	-	MR-proADM	8
Van Oers 2021 [46]	The Netherlands	2020	105	In-hospital mortality	Prospective observational study	30	72	20%	75	65	25	MR-proADM	8
Cheng 2020 [47]	China	2020	89	In-hospital mortality	Retrospective observational study	36	69	44%	53	54	45%	Amyloid A	7
Tufa 2022 [48]	Ethiopia	2021	126	Disease severity	Comparative cross-sectional study	68	60	57%	58	32	57%	Amyloid A	7
Harouni 2021 [49]	Egypt	2020	150	Disease severity	Case-control observational study	52	50	37%	98	48	43%	Amyloid A	8
Soltane 2023 [50]	Egypt	2021	273	Disease severity	Cross-sectional observational study	39	62	33%	177	43	36%	miR-155	8
Gaytan 2022 [51]	Mexico	2022	41	Disease severity	Cross-sectional observational study	41	53	15%	42	39	55%	miR-155	7
Kadhim 2024 [52]	Iraq	2023	105	Disease severity	Case-control observational study	35	46	37%	35	46	37%	miR-155	8
Karsli 2022 [53]	Turkey	2022	100	Disease severity	Prospective case-control study	60	66	-	40	64	-	Galectin-3	8
Kartal 2021 [54]	Turkey	2021	176	Disease severity	Cross-sectional observational study	64	-	-	72	-	-	Galectin-3	8
Vasbinder 2024 [55]	United States	2022	1962	In-hospital mortality	Prospective observational study	-	-	-	-	-	-	SuPAR	8
Vasiliou 2022 [56]	Greece	-	95	In-hospital mortality	Prospective observational study	-	-	-	-	-	-	SuPAR	8
Arnold 2021 [57]	United Kingdom	2020	187	Disease severity	Prospective cohort study	39	66	44%	148	55	47%	SuPAR	7
Chalkias 2022 [58]	Greece, Spain, and Denmark	2021	767	Disease severity	Prospective observational study	154	-	-	613	64	-	SuPAR	8
Molfino 2024 [59]	Italy	2021	93	In-hospital mortality	Prospective observational study	21	-	-	72	-	-	SuPAR	8

NLR: neutrophil-to-lymphocyte ratio, KL-6: Klebs von den Lungen-6, MR-proADM: Mid-Regional pro-Adrenomedullin, suPAR: soluble urokinase Plasminogen Activator Receptor, miR: microRNA.

**Table 2:** ROC analysis for outcome prediction according to baseline biomarker values.

Biomarker	Study	Outcome	AUC (95% CI)	Cut-off	Sensitivity (%)	Specificity (%)
NLR	Ardestani 2022 [23]	In-hospital mortality	0.726 (0.674–0.806)	6.830	82.8%	56.2%
NLR	Arini 2024 [24]	Disease severity	-	3.8	95%	74%
NLR	Bohra 2022 [26]	Disease severity	-	6.44	85.7%	96.8%
NLR	Hammad 2021 [27]	Disease severity	0.98	3.1	91%	93%
NLR	Basoglu 2020 [28]	Disease severity	0.834 (0.766–0.902)	2.8	75%	74%
NLR	Herrera 2021 [29]	Disease severity	0.98	3.1	91%	93%
NLR	Saya 2021 [30]	Disease severity	0.831	7.4	75%	84%
NLR	Tufa 2022 [31]	In-hospital mortality	0.75 (0.60–0.91)	4.63	86.7%	55.9%
KL-6	Alessandro 2021 [32]	Disease severity	0.85	455	75%	80%
KL-6	Alessandro 2020 [33]	Disease severity	0.85	455	75%	80%
KL-6	Bergantini 2021 [34]	Disease severity	0.95	-	85.7%	85.7%
KL-6	Maruyama 2022 [35]	Disease severity	0.89 (0.83–0.96)	966	81.6%	84.3%
MR-proADM	Garcia 2021 [37]	In-hospital mortality	0.832	0.80	96.9%	58.4%
MR-proADM	Gregoriano 2021 [38]	In-hospital mortality	0.78	0.93	92.9%	60%
MR-proADM	Cameli 2023 [39]	In-hospital mortality	0.958	1.02	82%	87%
MR-proADM	GarciaDe 2021 [40]	In-hospital mortality	0.905 (0.829–0.955)	1.01	85.7%	84.7%
MR-proADM	Montmollin 2022 [41]	In-hospital mortality	0.744	1	77.5%	68.8%
MR-proADM	Mangioni 2022 [42]	In-hospital mortality	0.87 (0.79–0.94)	1.04	92.3%	75%
MR-proADM	Popov 2022 [44]	In-hospital mortality	-	0.895	75%	69%
MR-proADM	Sozio 2021 [45]	In-hospital mortality	0.849	0.895	85.7%	68.7%
MR-proADM	vanOers 2021 [46]	In-hospital mortality	0.84 (0.76–0.92)	1.57	88%	67%
Amyloid A	Cheng 2020 [47]	In-hospital mortality	0.947	183.6	96.1%	94.3%
Amyloid A	Tufa 2022 [48]	Severity	0.703 (0.611–0.794)	1074	5.9%	98.3%
miR-155	Soltane 2023 [50]	Severity	0.927	-	96.6%	87.3%
miR-155	Kadhim 2024 [52]	Severity	0.91	4.06	82%	88%
Galectin-3	Karsli 2022 [53]	Severity	0.701 (0.582–0.819)	11.3	75%	50%
Galectin-3	Kartal 2021 [54]	Severity	0.89 (0.83–0.94)	18.9	87%	73%
suPAR	Vasbinder 2024 [55]	Severity	0.712	4	95.4%	11.8%
suPAR	Vassiliou 2022 [56]	Severity	0.81 (0.71–0.91)	6.3	74.2%	85.9%
suPAR	Arnold 2021 [57]	Severity	0.81 (0.72–0.88)	5.2	82%	65%

AUC: Area Under Curve, NLR: neutrophil-to-lymphocyte ratio, KL-6: Klebs von den Lungen-6, MR-proADM: Mid-Regional pro-Adrenomedullin, suPAR: soluble urokinase Plasminogen Activator Receptor, miR: microRNA.

## Data Availability

No new data were created.
